# Food allergy among university students: uncharted territory

**DOI:** 10.1186/s13223-020-0415-5

**Published:** 2020-03-04

**Authors:** Ali Hassan, Amna Alsaihati, Malak Al Shammari, Haitham Alaithan, Wejdan Al-Johani, Nouf AlShamlan, Salman Aljubran

**Affiliations:** 1 Department of Internal Medicine, King Fahd Hospital of the University–Imam Abdulrahman Bin Faisal University, Al-Khobar, Saudi Arabia; 2 Department of Family and Community Medicine, Imam Abdulrahman Bin Faisal University, Dammam, Saudi Arabia; 3 Department of Allergy and Immunology, Children’s Mercy Hospital, University of Missouri-Kansas City School of Medicine, Kansas City, MO USA

**Keywords:** Food allergy, University students, High-risk behavior, Saudi Arabia

## Abstract

**Background:**

Food allergy is a growing global health concern, with limited studies conducted in developing countries. High-risk behavior regarding allergies is common among young adults, making them prone to severe allergic reactions. This study aimed to estimate the prevalence of food allergies among Imam Abdulrahman Bin Faisal University (IAU) students and to determine the rate of high-risk behaviors in this group.

**Methods:**

An online survey was conducted among IAU students enrolled between the academic years of 2008 and 2018. The survey addressed food allergies with respect to age of onset, allergy testing, self-injectable epinephrine (SIE) prescription, associated allergic conditions, and attitude and behavior of university students regarding food avoidance and epinephrine use.

**Results:**

In total, 5497 completed surveys were analyzed; 526 participants were clinically diagnosed with allergies to at least one food item. A SIE device was prescribed for only 129 (24.5%) of the diagnosed individuals, of which only 4.7% carried the device at all times. Thirty-nine individuals (30.2%) reported that they and their family members know the proper epinephrine device technique. Strict avoidance of food allergens was reported by 30.2% of the individuals. Associated allergic conditions were observed in 69.3% of the clinically diagnosed individuals.

**Conclusion:**

Food allergies are prevalent among IAU students, with under-prescription of SIE devices. A high rate of high-risk behaviors with respect to food avoidance and carrying SIE was noted. Interventional strategies are needed to mitigate the risk of severe reactions among these university students.

## Background

The prevalence of food allergies (FA) is increasing worldwide [1]. In the United States, the prevalence of FA is 8% in children and 10.8% among adults [2, 3]. The exact prevalence of FA in Saudi Arabia is not known. Only a few studies have been conducted to evaluate FA in Saudi Arabia. In 1998, El-Rab conducted a study of 217 patients with a history of asthma, rhinitis, or urticaria and found that 17.5% had detectable levels of immunoglobulin E (IgE) specific for various food allergens [4]. A study of 1341 asthmatic patients in 2000 reported that 29% of the participants exhibited allergic symptoms to foods [5].

FA among university students presents a uniquely challenging situation, since many young adults become independent and unsupervised during these years. They are now responsible for their own health, including deciding which foods to eat and whether to take or carry medications. Young adults are more likely to take risks with regard to FA, placing them at higher risk for severe food-induced allergic reactions [6]. Unfortunately, universities lack a well-established supportive system for students with FA [7]. A survey-based cross-sectional study of undergraduate students at the University of Michigan reported that 57% of respondents (293/513) had FA and 36% of these allergic students experienced symptoms consistent with anaphylaxis. Moreover, risk-taking behavior was common in this cohort. Only 6.6% always carried self-injectable (SIE) device, while only 40% avoided the food allergen all the time [7]. In two case series, a total of 63 deaths due to anaphylaxis secondary to food were reported; 18 (28.5%) of these patients were between the ages of 18 and 25 years, and half of these deaths occurred in university or school settings [8, 9]. These findings indicate that severe or life-threatening allergic reactions to food are more frequently observed in older adolescents and young adult patients.

This study aimed to determine the prevalence of FA among students at the Imam Abdulrahman Bin Faisal University (IAU) in the Eastern Province of Saudi Arabia and to evaluate the frequency of risk-taking behaviors in this group. The results of this study may serve as a resource for developing interventional strategies to address FA-related risk and management behaviors.

## Methods

### Study design

This cross-sectional study was designed to assess the prevalence of FA in a sample of current and former students at IAU and to evaluate the frequency of risk-taking behaviors in this group. The study was approved by the Institutional Review Board of IAU. A cover letter that described the purpose of the study, voluntary nature of participation, and contact information was provided along with the survey. Participants were encouraged to contact the investigators if they had any queries regarding the study.

A 3-minute online survey was designed using QuestionPro survey software (Seattle, WA, USA). The survey included 21 questions and was available in both Arabic and English (Table 1). The survey addressed FA with respect to age of onset, emergency department visits, allergy testing, SIE prescription, the need to use the SIE, associated allergic conditions, and attitude and behavior of university students regarding food avoidance and epinephrine use as well as the social impact of FA. There were questions asking about the food allergens and the associated allergic symptoms; the results of these questions are outside the scope of the current paper and will be reported separately.

**Table 1:** English version of the food allergy questionnaire used in the study

No.	Question
1	Are you a current/former student of the Imam Abdulrahman Bin Faisal University?
2	What is your age?
3	What is your gender?
4	Where do you study?
5	Have you ever noticed any of the symptoms while consuming any specific food? Choices: abdominal pain/diarrhea/hives/vomiting/difficulty breathing/cough/swelling og lips and tongue/chest pain/wheezing/fainting
6	Have you noticed any of these symptoms again when you eat that specific food?
7	Which of the following food items cause your symptoms? Choices: eggs/milk/fish/tree nuts/shellfish/peanuts/soy/wheat/other (specify)
8	At what age did you start having these symptoms?
9	Do you think you have food allergy?
10	Have you been seen by a doctor for these symptoms?
11	Have your symptoms been diagnosed as food allergy by a physician?
12	Was the diagnosis of your food allergy confirmed by an allergy test (e.g., skin prick test)?
13	Have you ever been to the Emergency Department for an allergic reaction after food ingestion?
14	Have you been prescribed an injection for allergies (e.g., EpiPen^®^)?
15	How often do you carry the injection for allergies? Choices: always/often/sometimes/rarely/never
16	Do you and your family members know how to use the injection correctly?
17	Have you ever had to use the injection?
18	How often do you avoid foods that may contain the allergen? Choices: always/often/sometimes/rarely/never
19	Does the food allergy restrict you from attending social events?
20	Have you been diagnosed with any of the following allergic conditions? Choices: atopic dermatitis/allergic rhinitis/asthma/allergic conjunctivitis/none
21	Do any of your first-degree relatives have a food allergy?

To avoid duplicate responses, participants were not allowed to respond to the survey more than once. We conducted a pilot study with a group of 100 students to assess the clarity of the questions and the time needed to complete the survey. After the pilot study, no major changes were made to the questions and the data from the pilot study were not included in the analyses.

### Study participants

Students enrolled at IAU between 2008 and 2018 were surveyed. A university database of addresses was used to obtain a total of 37,103 mobile numbers registered to students. The survey commenced on June 8, 2019 and was open for a total of 14 days. A personalized message with a link to the online survey was sent to 34,064 students whose mobile numbers were registered with the WhatsApp application (Facebook, Menlo Park, CA, USA). Three days later, a reminder message was sent to those who had not completed the survey. The participants were encouraged to share the survey via social media to reach their fellow university students.

### Statistical analysis

The collected data were compiled using the QuestionPro platform and analyzed using IBM SPSS for Windows, Version 25 (IBM Corp., Armonk, NY, USA). Descriptive statistics, such as percentages and frequency distributions of different characteristics, were used as appropriate. The Chi square test was used to compare the categorical variables. Statements of statistical significance were based on significance level of α = 0.05.

## Results

Of the 34,064 students who received an invitation to participate in the survey, 6050 (17.8%) completed the survey. After review, the 553 participants identified as non-IAU students were excluded. Of those remaining, 5497 individual survey results were deemed valid and included in further analyses.

### Characteristics of the participants

The study included 3535 female and 1962 male participants, representing 64.3% and 35.7% of the study population, respectively. The average age of the participants was 22.60 ± 4.96 years. Participants were recruited from various colleges within the university, including the Colleges of Health, Engineering, Science and Management, and the Colleges of Arts and Education (Table 2).

**Table 2:** Gender and college distribution of participants

College	Gender	N	(%)
Colleges of Health	Male	685	(12.5)
	Female	921	(16.8)
Colleges of Engineering	Male	324	(5.9)
	Female	142	(2.6)
Colleges of Science and Management	Male	710	(12.9)
	Female	1581	(28.8)
Colleges of Arts and Education	Male	115	(2.1)
	Female	807	(14.7)
Preparatory year	Male	128	(2.3)
	Female	84	(1.5)

*N* number of participants

### Prevalence of FA

Of the 5497 participants, 526 (9.6%), including 155 males and 371 females, had a clinical diagnosis of FA, and 284 reported having allergies to more than one food item. Furthermore, 174 (33.1%) of those patients with clinically diagnosed FA had undergone allergy evaluations to confirm their diagnoses (Table 3). No statistically significant difference was observed in the prevalence of FA in terms of gender. Among participants with clinically diagnosed FA, 58% reported having a first-degree relative with FA. Overall, 275 (52.3%) reported visiting the emergency department at least once because of symptoms related to FA. A total of 443 (8.1%; 138 male and 305 female participants) believed that they had undiagnosed FA. Most of these participants (83.3%) did not report experiencing symptoms that necessitated a visit to the emergency department.

**Table 3:** Subgroup analysis for food allergies confirmed by allergy testing

	Clinical diagnosis of FA (N = 526)	Confirmed FA (N = 174)	X^2^ (*P* value^a^)
SIE prescription	129 (24.5%)	72 (41.4%)	*9.46 (0.002)*
Required to use SIE	58 (45.0%)	34 (47.2%)	0.03 (0.85)
Always carrying SIE	6 (4.7%)	6 (8.3%)	0.98 (0.32)
Know how to use SIE	39 (30.2)	30 (41.7%)	1.28 (0.26)
ED Visit due to FA	275 (52.3%)	103 (59.2%)	0.74 (0.39)
Strict avoidance of allergens	159 (30.2%)	63 (36.2%)	1.09 (0.30)
Social restrictions due to FA	121 (23.0%)	42 (24.1%)	0.06 (0.81)
Family history of FA	305 (58.0%)	104 (59.8%)	0.04 (0.83)
Associated allergic conditions	320 (60.8%)	127 (73.0%)	1.78 (0.18)

*N* number of participants, *ED* emergency department, *FA* food allergies, *SIE* self-injectable epinephrine

^a^P-value in italics when significant

### Age of onset of FA

Among the participants known to have FA, 51.7% reported an onset during childhood (≤ 13 years). Adolescent (14–17 years) and adult-onset (≥ 18 years) FA were reported by 29.1% and 19.2% of participants, respectively.

### Self-injectable epinephrine

Among the participants with clinically diagnosed FA, 129 (24.5%) were prescribed SIE (e.g., EpiPen^®^). However, only 39 of these 129 participants (30.2%) reported that they and their family know the correct way of using SIE. Furthermore, 58 (45%) participants had to use SIE. Participants who had a severe allergic reaction secondary to food ingestion requiring an emergency department visit were more likely to be given SIE prescription (P < 0.05). The majority of participants (79.9%) who were prescribed SIE reported that they never or rarely carried it; only 4.7% reported that they always carried it with them. Participants who needed to use SIE were more likely to carry one with them at all times (P = 0.032).

### Avoidance of allergens

Only 159 (30.2%) participants with clinically diagnosed FA reported that they always avoided food allergens. Furthermore, participants who were prescribed SIE and/or were diagnosed with FA during childhood were more likely to report strict avoidance of the allergens (P < 0.05).

### Social impact of FA

Nearly a quarter of the participants clinically diagnosed with FA indicated that a FA restricted them from attending social events. This restriction was reported more frequently by female participants (14.4%) than by male participants (8.6%) (P = 0.035).

### Associated allergic conditions

Of the 526 participants clinically diagnosed with FA, 69.3% reported associated allergic conditions, and female participants were more likely than male participants to report concomitant allergic conditions. Atopic dermatitis was the most prevalent allergic condition and was reported by 37.1% of the participants with clinically diagnosed FA. A statistically significant association was observed between atopic dermatitis and FA (P < 0.05). Other allergic conditions, such as asthma, allergic rhinitis, and allergic conjunctivitis, were observed in 18.8%, 18.1% and 14.2% of participants with clinically diagnosed FA, respectively. Participants with multiple FA were more likely to have associated atopic dermatitis (P = 0.002) or allergic conjunctivitis (P = 0.015).

## Discussion

This cross-sectional survey-based study of IAU students revealed that 9.6% of the respondents were allergic to at least one food item based on a clinical diagnosis. This finding is consistent with other studies of adults with FA. In an earlier survey of more than 40,000 adults in the United States, Gupta et al. estimated a FA prevalence of 10% [3].

In our study, 8.1% of the participants reported a perceived but undiagnosed food allergy because they had experienced recurrent unpleasant symptoms following the ingestion of a specific food. Gupta et al. reported that the number of adults who believed that they had a food allergy was nearly twice as high as the number of those with an actual food allergy [3].

Most people with FA develop them during childhood, though FA can develop at any age. In our study, 19.2% of students reported adult-onset FA. Similarly, in a retrospective study of more than 1000 patients with FA by Kamdar et al., at least 15% reported adult-onset FA [10]. Interestingly, Kamdar et al. found that patients with adult-onset FA faced a higher risk of severe reactions [10].

Nearly 70% of students with clinically FA in our study reported having other allergic diseases; atopic dermatitis was the most common one, affecting 37.1% of students with FA. Studies of patients with atopic dermatitis have reported a food sensitization prevalence of 66%, with rates as high as 81% for the prevalence of challenge-proven FA [11]. Furthermore, asthma is a risk factor for the development of fatal food-induced allergic reactions [8, 9, 12].

Epinephrine injection is the treatment of choice for food-induced anaphylaxis, and all patients with a diagnosed FA should receive a prescription for SIE [13]. However, we found that only a quarter of students with clinically diagnosed FA were prescribed SIE and those who had a visiting to the emergency department because of FA were more likely to have received a prescription. These results were consistent with those of a recent population-based survey of adults with FA in the United States [3]. Moreover, the SIE prescription rate was significantly higher among participants whose FA was confirmed by allergy testing. We believe that the low prescription rate in our study is attributable to the limited availability of SIE in Saudi Arabia. In a 2003 study, Simons studied the availability of SIE for patients at a risk of anaphylaxis in different countries and found that auto-injectors containing doses appropriate for adults were available in only 56.4% of these countries [14]. A follow-up study in 2007 revealed a slight improvement, as 59% of the participating countries reported the availability of SIE with adult doses [13].

SIE device can be lifesaving when used correctly. However, patients must always carry the device, given the unpredictable severity of allergic reactions [3]. Most fatalities due to FA occur when patient does not have access to epinephrine at the time of a reaction or when epinephrine administration is delayed [8, 9]. In this study, only 4.7% reported that they always carried SIE with them, and these students were more likely to report experiencing a prior event requiring the use of epinephrine. In a previous survey-based study of 174 adolescents and young adults, 39% reported that they did not carry epinephrine at all times particularly when participating in sports or when wearing tight clothing, and 38% reported they did not have epinephrine at the time of their most recent reaction [6]. Another study of 188 adolescents with FA at 2 allergy clinics in England reported that only 16% always carried epinephrine and strictly avoided the food allergen [15]. The rate of students who always carried SIE in a study at the University of Michigan was comparable to that of our study [7]. Even after implementing a comprehensive FA support program, however, a follow-up of the University of Michigan study demonstrated only modest improvement [16]. Since only one-third of participants with clinically diagnosed FA underwent allergy testing for confirmation, we first postulated that the risk-taking behavior in the study might be overestimated due to the presence of large number of participants who may had food intolerance and were misdiagnosed as FA. However, we performed subgroup analysis involving only participants with confirmed FA. Interestingly, the analysis confirmed our original findings.

In our study, 30% of students reported strict avoidance of the food allergen. Notably, students who were prescribed SIE were more likely to report strict allergen avoidance. Again, this rate was comparable to that reported by the study of students with FA at the University of Michigan, where 40% reported strict allergy avoidance [7]. Another study of adolescents and young adults with FA revealed that 42% had consumed foods containing the allergen, despite the presence of a precautionary warning on food labels [6]. Notably, the same study indicated that individuals who displayed high-risk behavior with respect to FA were more likely to have experienced a recent allergic reaction and were more likely to report feeling “different” because of their FA [6].

The risk of a severe allergic reaction and the anxiety associated with the strict avoidance of food allergens have been shown to negatively impact the quality of life of these patients [17]. In our study, a quarter of the overall students with FA, including a larger proportion of female students, reported that their FA restricted them from attending social events.

The present study had limitations, given the fact it was a survey-based study. The participants’ FA were self-reported. Although self-reporting can be rapid and convenient, it may introduce some bias. We have not performed objective allergy testing on the participants with clinically diagnosed as having FA. Further studies based on a formal medical investigation are needed to better define the true prevalence and patterns of FA in university students.

## Conclusion

FA is a prevalent health problem among university students enrolled in IAU between 2008–2018. There is a high rate of risk-taking behavior with respect to food avoidance and carrying SIE. Interventional strategies are needed to mitigate the risk of severe reactions among these university students.

## Data Availability

The datasets used and/or analyzed during the current study are available from the corresponding author on reasonable request.

## Abbreviations

FA: Food allergy
IAU: Imam Abdulrahman Bin Faisal University
SIE: Self-injectable epinephrine
